# Distinct humoral responses induced by heterologous versus homologous prime–boost immunization strategies in early life

**DOI:** 10.3389/fimmu.2025.1563345

**Published:** 2025-08-07

**Authors:** Poorya Foroutan Pajoohian, Audur Anna Aradottir Pind, Jenny Lorena Molina Estupiñan, Dennis Christensen, Gabriel Kristian Pedersen, Thorunn A. Olafsdottir, Ingileif Jonsdottir, Stefania P. Bjarnarson

**Affiliations:** ^1^ Faculty of Medicine, Biomedical Center, School of Health Sciences, University of Iceland, Reykjavik, Iceland; ^2^ Department of Immunology, Landspitali, the National University Hospital of Iceland, Reykjavik, Iceland; ^3^ Center for Vaccine Research, Statens Serum Institut, Copenhagen, Denmark

**Keywords:** neonatal vaccination, heterologous immunization routes, antibody-secreting cells, antibody levels, mucosal immunity

## Abstract

An important component in the development of efficacious vaccines is the optimization of vaccination schedules to elicit protective immunity, especially in a vulnerable group like infants. Mucosal IgA plays an important role in the initial defense at mucosal surfaces, protecting against the colonization of respiratory pathogens, potentially reducing invasion and disease severity, while systemic immunity relies on protective IgG antibodies (Abs). This study aims to design an immunization strategy inducing both optimal systemic and mucosal immune responses using heterologous prime–boost immunization in comparison to homologous immunization. We immunized neonatal mice with a pneumococcal conjugate vaccine, Pn1-CRM_197_, by heterologous subcutaneous (s.c.) priming with CAF01 adjuvant, followed by intranasal (i.n.) booster with multiple mutant of cholera toxin (mmCT) adjuvant or homologous immunizations by either route. Schedules including mucosal immunization, either once in heterologous s.c. priming followed by i.n. booster or twice with homologous i.n./i.n. immunization, induced higher serum and mucosal vaccine-specific IgA Abs and Ab-secreting cells (ASCs) than homologous s.c./s.c. immunization. However, heterologous s.c./i.n. immunization did not induce vaccine-specific IgG Abs in serum and the lung to a comparable level with that of homologous s.c./s.c. immunization. The immunization route in priming and boosting affected the induction of specific ASCs in lymphoid organs. Homologous s.c./s.c. immunization induced systemic (spleen) and local (inguinal lymph nodes) IgG responses. Homologous i.n./i.n. immunization induced systemic and local mucosal IgA responses, observed by enhanced salivary and lung IgA Abs. Meanwhile, heterologous s.c/i.n. immunization induced local humoral IgG and IgA responses in draining lymph nodes (cervical) and accelerated the early homing of IgG and IgA ASCs to the bone marrow compared to homologous i.n./i.n. immunization. Increasing the vaccine dose in the i.n. booster of heterologous immunization was needed to improve further humoral immune responses. Taken together, homologous s.c./s.c. immunization induces higher systemic IgG responses than heterologous s.c./i.n., which could be enhanced by increasing the vaccine dose in the i.n. booster but was still lower than after s.c./s.c. immunization. However, heterologous s.c./i.n. immunization has the advantage of inducing IgA responses, comparable to homologous i.n./i.n. immunization. This study indicates that heterologous immunization schedules may be an attractive approach for inducing early-life systemic and mucosal humoral immune responses.

## 1 Introduction

Vaccines have a significant impact on public health, saving 2–3 million lives worldwide each year (1). Despite considerable progress in vaccination efforts, a significant proportion of children under 5 years of age die from infectious diseases. Almost half of the 5.3 million global deaths in this age group are caused by infections, underscoring the urgency for further advancements in vaccination and increased access to existing vaccines (2). Poor antibody (Ab) responses are among the hallmarks of neonatal immune responses, requiring multiple doses to establish immunological memory and achieve sustained protective immunity (3). Several factors are involved in this limitation including attenuated B-cell germinal center (GC) reactions due to underdeveloped follicular dendritic cells (FDCs) (4), preferential differentiation into memory B cells rather than plasmablasts (5), and insufficient survival signals leading to the reduced survival of long-lived plasma cells in the bone marrow (BM) (6, 7). The strategic development of vaccination schedules, e.g., by incorporating adjuvants into vaccine formulations and using various routes of immunization for priming and boosting, can enhance humoral and cellular immune responses (8–13). In conventional prime–boost strategies, typically the vaccine is administered by the same route multiple times (homologous prime–boost immunization). Progress in vaccination science suggests that using diverse vaccine formulations and adjuvants along with different delivery systems with the same or different forms of the antigen (heterologous prime–boost immunization) can enhance immunogenicity (14). Furthermore, it has been shown that the route of immunization significantly shapes the immune response: parenteral administration leads to high serum IgG levels, and intranasal vaccination induces IgA responses in both serum and intestinal lavage (15). Systemic IgG responses have been demonstrated to be the most effective in fighting invasive diseases caused by encapsulated bacteria like *Streptococcus pneumoniae* (16, 17). IgA responses, particularly at mucosal sites, can play a role in preventing carriage (18) and spreading infections (19, 20). Adjuvants are important in enhancing, modulating, and prolonging immune responses (21, 22). Currently, alum is used as an adjuvant in most vaccines licensed for human infants, but MF-59 is approved for H1N1 influenza vaccination in infants aged 6 months and older (23–25). New adjuvants have been shown to overcome limitations of early-life immunity (26, 27). Our group has shown that adjuvants such as LT-K63, MF-59, multiple mutant of cholera toxin (mmCT), and IC31, administered subcutaneously (s.c.) with a pneumococcal conjugate vaccine (Pnc1-TT) to neonatal mice can enhance GC induction, elevate Ab responses, and improve protection against bacteremia and lung infections (9, 28–30) in a well-established neonatal mouse model of vaccination and pneumococcal challenge (13, 31–33). We have shown that the potent mucosal and systemic adjuvant LT-K63 co-administered with Pnc1-TT induces protective immunity against lethal pneumococcal infections in neonatal mice and that intranasal (i.n.) immunization was superior to s.c. immunization, especially after a single immunization (9). Thus, different adjuvants, vaccines, and routes of administration can highly improve early-life immune responses. In the present study, the aim was to compare heterologous and homologous prime–boost immunization route strategies in overcoming the limitation of humoral immune responses, including vaccine-specific IgG and IgA Abs and Ab-secreting cells (ASCs) in early life, using the adjuvants CAF01 and mmCT with a pneumococcal conjugate vaccine (Pn1-CRM_197_). CRM_197_ is a genetically detoxified diphtheria toxin (DT) that contains a single amino acid substitution from glycine to glutamate in position 52 (51). CRM_197_ has been identified as an ideal carrier protein for conjugate vaccines, enabling T cell-dependent immune responses to poorly immunogenic capsular polysaccharides, especially in infants (reviewed in (52)). CAF01 is composed of a liposomal delivery vehicle formed by the cationic surfactant dimethyldioctadecylammonium (DDA) incorporating the immunostimulator trehalose 6,6′-dibehenate (TDB). CAF01 signals via the C-type lectin receptor (CLR) Mincle and activates the Syk/Card9 pathway and the production of pro-inflammatory cytokines (34–36). In adult mice and humans, CAF01 elicits strong TH1/TH17 responses (10, 37), and in early life, it enhances germinal center reactions after a single dose when combined with hemagglutinin (HA) or Respiratory Syncytial Virus (RSV) pre-F antigen vaccines (27, 38). Cholera toxin (CT) is a potent mucosal adjuvant that cannot be used in human vaccines due to toxicity; however, inactive mutants of CT, including the mmCT, possess adjuvant effects while exhibiting low or no toxicity (39). mmCT has been shown to enhance neonatal immune responses when given s.c. with Pnc1-TT and TT (29, 40). Additionally, when administered to neonatal mice i.n. with Pn1-CRM_197_, mmCT enhanced GC activation, vaccine-specific serum IgG and IgA, along with salivary IgA, and vaccine-specific IgG and IgA ASCs in the spleen and BM, leading to both the enhanced induction and persistence of the immune response (12). Furthermore, mmCT has been shown in adult mice to increase serum IgG, mucosal IgA responses, and CD4 T-cell responses, especially Th17 cells, after intragastric immunization with a whole-cell inactivated *Helicobacter pylori* vaccine (41, 42). In this study, we demonstrated that CAF01 and mmCT in the heterologous and homologous prime–boost immunization route schedules can enhance vaccine-specific humoral immune responses in neonatal mice, although in a different manner. Although there were clear benefits of i.n. immunization, either once in heterologous s.c./i.n. immunization or twice in homologous i.n./i.n., in terms of vaccine-specific IgA responses, we demonstrated that in order to elicit IgG Abs almost to a comparable level to those elicited by homologous s.c./s.c. immunization, the dose of the vaccine Pn1-CRM_197_ had to be increased when administered i.n. in the heterologous prime–boost immunization route strategy.

## 2 Materials and methods

### 2.1 Mice

Naval Medical Research Institute (NMRI) mice (5–6 weeks) from Taconic (Skensved, Denmark) were mated and housed in microisolator cages at the ArcticLAS vivarium facility (Reykjavík, Iceland), with controlled temperature, light, and humidity, and provided with standard food and water *ad libitum*. Breeding cages were monitored daily, and newborn pups remained with their mothers until weaned at 4 weeks old. The experimental protocol was approved by the Experimental Animal Committee of Iceland under regulations 279/2002.

### 2.2 Antigen, adjuvants, and immunization

The pneumococcal conjugate vaccine Pn1-CRM_197_, provided by the Serum Institute of India (Pune, India), consists of pneumococcal polysaccharide of serotype 1 (Pn1) conjugated to a genetically detoxified mutant of diphtheria toxin (CRM_197_), which serves as a carrier protein (43), with the ratio 1.1 of carrier (CRM_197_) to polysaccharide (Pn1). In this study, Pn1-specific immune responses for IgG and IgA antibodies/antibody-secreting cells were measured. The adjuvant mmCT, produced as previously described (39), is a non-toxic mutant of CT that retains much of the adjuvant activity of native CT. It is generated by genetically modifying the enzymatically active A subunit (CTA) to resist site-specific proteolytic cleavage (“nicking”), which is required for its toxicity. This resistance is achieved through multiple mutations that protect the protein from cleavage by *Vibrio cholerae* proteases. The adjuvant was provided by the Gothenburg University Vaccine Research Institute (GUVAX) (Gothenburg, Sweden). CAF01 (250 µg dimethyldioctadecylammonium and 50 µg trehalose dibehenate) was produced as previously described (44) by incorporating the glycolipid TDB into cationic liposomes composed of the quaternary ammonium compound DDA. CAF01 induces a strong cell-mediated immune response as well as a robust antibody response. It was provided by Statens Serum Institut (Copenhagen, Denmark). The vaccine solutions were prepared 1 h before immunization. For s.c. priming, 50 µL of vaccine solution (neonatal mice) and, for s.c. booster immunization, 100 µL of vaccine (infant mice) were injected on either side of the base of the tail. For i.n. immunization, 2 × 2.5 µL of vaccine solution for priming (neonatal mice) and two 2 × 3 µL for booster (infant mice) were slowly delivered into the nares, with 30 min between doses. Anesthesia was given before i.n. booster.

### 2.3 Blood, saliva, and lung homogenate sampling

Mice were bled from the tail vein at 2 weeks post-priming and weekly for 1 to 5 weeks post-booster to measure Pn1-specific IgG and IgA in serum. Saliva was collected using absorbent sticks inserted into the mouth for 5 min and then transferred to Phosphate Buffered Saline (PBS) with 10.0 µg/mL protease inhibitor (aprotinin; Sigma-Aldrich, St. Louis, MO, USA) to prevent proteolysis. Pooled saliva samples per group were stored at −70°C. At each experimental endpoint, the lungs were perfused with 5 mL PBS via the heart, gently washed in PBS, transferred to PBS with 10.0 µg/mL protease inhibitor (aprotinin; Sigma-Aldrich, St. Louis, MO, USA) to prevent proteolysis, and then homogenized using a homogenizer to collect lung homogenates for measuring anti-Pn1 IgA and IgG levels.

### 2.4 Enzyme-linked immunosorbent assay

Anti-Pn1 IgG and IgA in serum, saliva, and lung homogenates were measured via enzyme-linked immunosorbent assay (ELISA) as described previously (9). Briefly, microtiter plates (MaxiSorp; Nunc AS, Roskilde, Denmark) were coated with 5 µg/mL of Pn1 (American Type Culture Collection, Rockville, MD, USA) in PBS for 5 h at 37°C, and the plates were washed and blocked with PBS with 0.05% Tween 20 (Sigma) containing 1% BSA (Bovine Serum Albumin) (Sigma). Serum samples and standards were neutralized with cell wall polysaccharide (CWPS; Statens Serum Institut), diluted 1:50 (1:25 for saliva and lung homogenates) in PBS with 0.05% Tween 20, and incubated in 500 mg/mL of CWPS for 30 min at room temperature. Neutralized serum samples and standards were serially diluted, whereas saliva samples were measured undiluted; both were incubated for 2 h in duplicates in 100 µL/well in Pn1-coated microtiter plates at room temperature. The plates were washed and then incubated with horseradish peroxidase-conjugated goat anti-mouse IgG or IgA (Southern Biotechnology Associates, Birmingham, AL, USA) diluted 1:5,000 in PBS-Tween 20 for 2 h at room temperature in 100 µL/well. The plates were washed, 3,3′,5,5′-tetramethylbenzidine substrate (Kirkegaard & Perry Laboratories, Gaithersburg, MD, USA) was used for development, and the reaction was stopped with 0.18 M H_2_SO_4_. Absorbance was measured at an optical density of 450 nm in a Multiskan FC Microplate Photometer (Thermo Scientific, Waltham, MA, USA). The standard for Pn1-specific Abs was prepared by pooling sera from adult mice hyperimmunized with the conjugate vaccine. The results were calculated from a standard curve and expressed as mean log of ELISA units (EU)/mL for IgG and mean of EU/mL for IgA.

### 2.5 Enzyme-linked Immunospot

Pn1-specific IgG and IgA ASCs in the spleen, lymph nodes (LNs), and BM were enumerated by enzyme-linked Immunospot (ELISpot), as previously described (28), at 2 and 5 weeks after booster. MultiScreen high protein binding immobilon-P membrane plates (Millipore Corporation, Bedford, MA, USA) were coated with 10 µg/mL Pn1 overnight at 37°C in 50 µL/well and blocked with complete Roswell Park Memorial Institute (RPMI) medium 1640 (Life Technologies BRL, Life Technologies, Paisley, UK). Duplicates of cells from the spleen and BM were incubated in four threefold dilutions starting with 1 × 10^7^ cells in 100 µL of complete RPMI 1640 per well for 5 h at 37°C, washed and incubated with alkaline phosphatase (ALP)-conjugated goat anti-mouse IgG or IgA (Southern Biotechnology Associates) overnight at 4°C, and developed by 5-bromo-4-chloro-3-indolyl phosphate and NBT in AP development buffer (Bio-Rad Labs, Hercules, CA, USA). The number of spots (each representing a cell secreting specific Abs) was counted using the ELISPOT reader ImmunoSpot^®^ S6 Ultimate using ImmunoSpot^®^ software [Cellular Technology Limited (CTL) Europe, Bonn, Germany].

### 2.6 Statistical analysis

Statistical analyses between immunization groups at each timepoint were first assessed using the Kruskal–Wallis test, and where significant differences were found, comparisons were performed using the Mann–Whitney U-test between two groups (*p ≤ 0.05, **p ≤ 0.01, and ***p ≤ 0.001), with p-values < 0.05 considered statistically significant. All analyses were conducted using GraphPad Prism 9.0 (GraphPad Software, La Jolla, CA, USA).

## 3 Results

### 3.1 Early-life immunization with intranasal booster results in higher vaccine-specific IgA responses than homologous subcutaneous prime–boost immunization

At the beginning of this study, it was necessary to determine the optimal dose of the Pn1-CRM_197_ vaccine. Thus, neonatal (7-day-old) mice were immunized twice with a 16-day interval s.c./s.c. or s.c./i.n. with 0.25, 0.5, or 0.75 of Pn1-CRM_197_ with or without CAF01 s.c. or mmCT i.n. It was found that the 0.25-μg dose of Pn1-CRM_197_ was optimal to assess the effects of adjuvants and immunization routes (**Supplementary Figures 1**, **2**) defined by the increase in Ab response when adding adjuvant to the vaccine formulation. Testing different doses of mmCT (1, 2, and 5 μg) showed that 2 μg was optimal (**Supplementary Figures 2**, **3**). Thus, 0.25 μg Pn1-CRM_197_, 2 μg mmCT, and CAF01 (250 µg dimethyldioctadecylammonium and 50 µg trehalose dibehenate) were used throughout the following experiments, except for the last result chapter, where increased doses of the vaccine in i.n. booster were assessed.

After the optimal dose of Pn1-CRM_197_ was determined, mice were immunized twice (7 and 23 days old) with 0.25 μg Pn1-CRM_197_ by heterologous or homologous prime/boost routes with or without CAF01 or mmCT, as follows: heterologous s.c. CAF01/i.n. mmCT (s.c./i.n.), homologous s.c. CAF01/s.c. mmCT (s.c./s.c.), or homologous i.n. mmCT/i.n. mmCT (i.n./i.n.). Control mice remained unimmunized, and two reference groups were included: 0.25 μg Pn1-CRM_197_ s.c./s.c. without adjuvant or with CAF01 in priming but no adjuvant in booster. In Section 3.3, the experimental mice were primed s.c. with 0.25 μg Pn1-CRM_197_ + CAF01 and received a booster i.n. with 0.25, 1, 2, or 4 μg Pn1-CRM_197_ + 2 μg mmCT.

**Figure 1:**
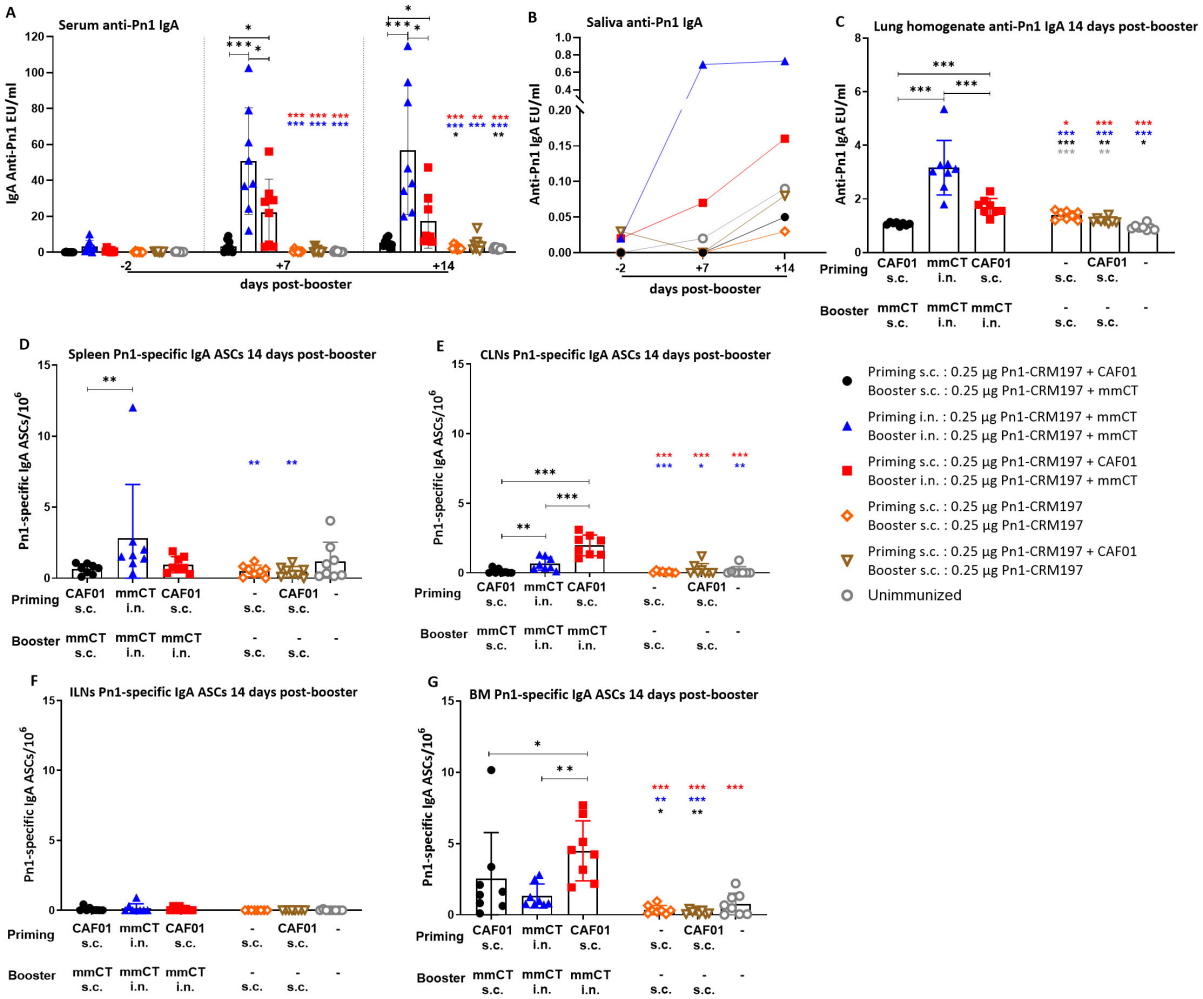
Early-life immunization strategies incorporating intranasal boosters result in superior anti-Pn1 IgA responses at 14 days post-booster. **(A)** Serum anti-Pn1 IgA levels at −2 to 14 days post-booster. **(B)** Saliva anti-Pn1 IgA levels at 7 to 14 days post-booster. **(C)** Lung homogenate anti-Pn1 IgA levels at 14 days post-booster. **(D–G)** Pn1-specific IgA^+^ ASCs in spleen, CLNs, ILNs, and BM at 14 days post-booster. Mice were immunized by different immunization schedules utilizing 0.25 µg of Pn1-CRM_197_, CAF01, and 2 µg of mmCT. Results are expressed as IgA levels (mean EU/mL ± SD) or number of spots/10^6^ cells (mean ± SD) in six to eight– mice per group. Statistical difference was calculated using Kruskal–Wallis test first (**A, C–E, G**, p < 0.0001), and then Mann–Whitney U-test was applied. *p < 0.05, **p < 0.01, and ***p < 0.001. Comparisons to reference groups are marked on the bars in the corresponding test group colors. ASCs, antibody-secreting cells; BM, bone marrow; mmCT, multiple mutant of cholera toxin.

**Figure 2:**
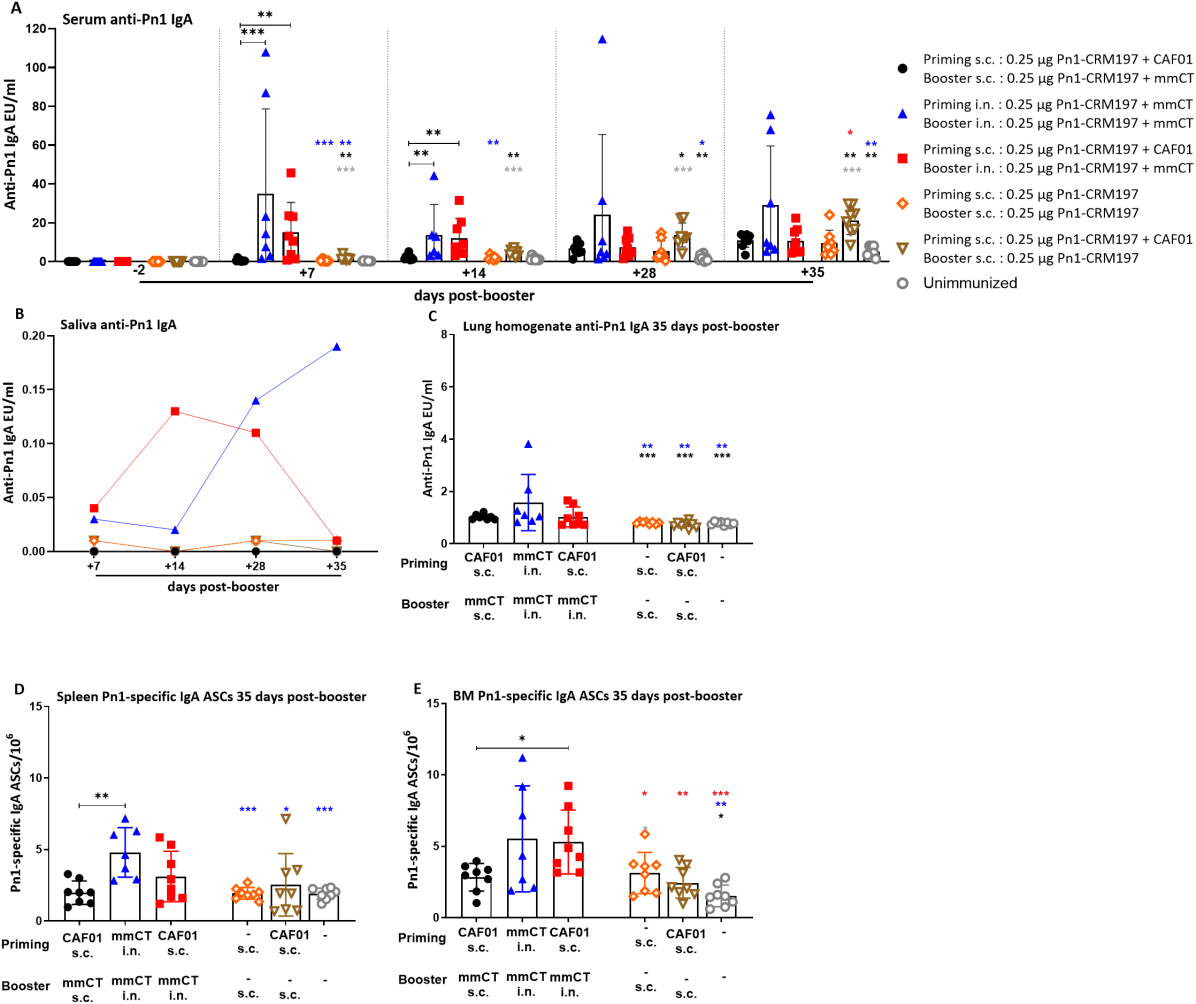
Early-life immunization strategies incorporating intranasal boosters result in superior anti-Pn1 IgA responses. **(A)** Serum anti-Pn1 IgA levels at −2 to 35 days post-booster. **(B)** Saliva anti-Pn1 IgA levels at 7 to 35 days post-booster. **(C)** Lung homogenate anti-Pn1 IgA levels at 35 days post-booster. **(D, E)** Pn1-specific IgA^+^ ASCs in spleen, CLNs, ILNs, and BM at 35 days post-booster. Mice were immunized by different immunization schedules utilizing 0.25 µg of Pn1-CRM_197_, CAF01, and 2 µg of mmCT. Results are expressed as IgA levels (mean EU/mL ± SD) or number of spots/10^6^ cells (mean ± SD) in six to eight– mice per group. Statistical difference was calculated using Kruskal–Wallis test first (**A, C–E**, p < 0.0001), and then Mann–Whitney U-test was applied. *p < 0.05, **p < 0.01, and ***p < 0.001. Comparisons to reference groups are marked on the bars in the corresponding test group colors. ASCs, antibody-secreting cells; BM, bone marrow; mmCT, multiple mutant of cholera toxin.

In order to assess the effects of the heterologous and homologous immunization schedules on mucosal humoral immune responses, we measured vaccine-specific IgA levels in saliva and lung homogenate along with systemic IgA in serum. Furthermore, we enumerated vaccine-specific IgA ASCs in the spleen, Cervical (CLNs) and inguinal (ILNs) lymph nodes, and bone marrow at 14 days post-booster (**Figure 1**) and also at 35 days post-booster to assess the persistence of the mucosal humoral immune response (**Figure 2**). Remarkably, we have previously shown that i.n. immunization without an adjuvant does not give any IgA or IgG Ab response (33).

Mucosal delivery by either schedule, once with heterologous s.c./i.n. or twice with homologous i.n./i.n. immunization, induced higher vaccine-specific IgA levels in serum, saliva, and lung homogenates compared to the homologous s.c./s.c. schedule at 7 to 35 days post-booster (**Figures 1A–C**, **2**). However, the salivary IgA response following heterologous immunization did not appear to be as persistent as after homologous i.n./i.n. immunization (**Figure 2B**). To assess whether the prime–boost immunization schedules with these adjuvants, CAF01 and/or mmCT, had different effects on the induction (day 14) of early-life vaccine-specific ASCs in the spleen and LNs along with their homing and persistence in the BM (day 35), we measured Pn1-specific IgA ASCs in the spleen, Cervical (CLNs) and inguinal (ILNs) lymph nodes, and BM at 14 and 35 days post-booster. Heterologous s.c./i.n. and homologous i.n./i.n. immunization induced comparable numbers of vaccine-specific IgA ASCs in the spleen at 14 and 35 days post-booster (**Figures 1D**, **2D**). The route of delivery had a clear effect on where the main induction was observed, as the number of specific IgA ASCs in the spleen was higher in homologous i.n./i.n. than homologous s.c./s.c. immunized mice (**Figure 1D**). However, heterologous s.c./i.n. immunized mice had a higher number of specific IgA ASCs in CLNs than both the homologous i.n./i.n. and s.c./s.c. schedules (**Figure 1E**), whereas in ILNs, no difference was observed between the different immunization schedules (**Figure 1F**). Importantly, heterologous s.c./i.n. immunization induced the highest number of specific IgA ASCs that had already homed to the BM at 14 days post-booster (**Figure 1G**) and persisted up to 35 days post-booster (**Figure 2E**), but at that later timepoint, their number was comparable to that in homologous i.n./i.n. immunized mice (**Figure 2E**).

Taken together, including i.n. immunization into an immunization schedule enhances the induction of IgA responses, whether it is only once as a heterologous s.c/i.n. or twice as homologous i.n./i.n. Furthermore, it seems that heterologous s.c./i.n. has some advantage in inducing IgA ASCs in draining lymph nodes and earlier homing to the bone marrow than homologous i.n./i.n. immunization.

### 3.2 Early-life homologous subcutaneous prime–boost immunization induces high levels of systemic vaccine-specific IgG responses

**Figure 3:**
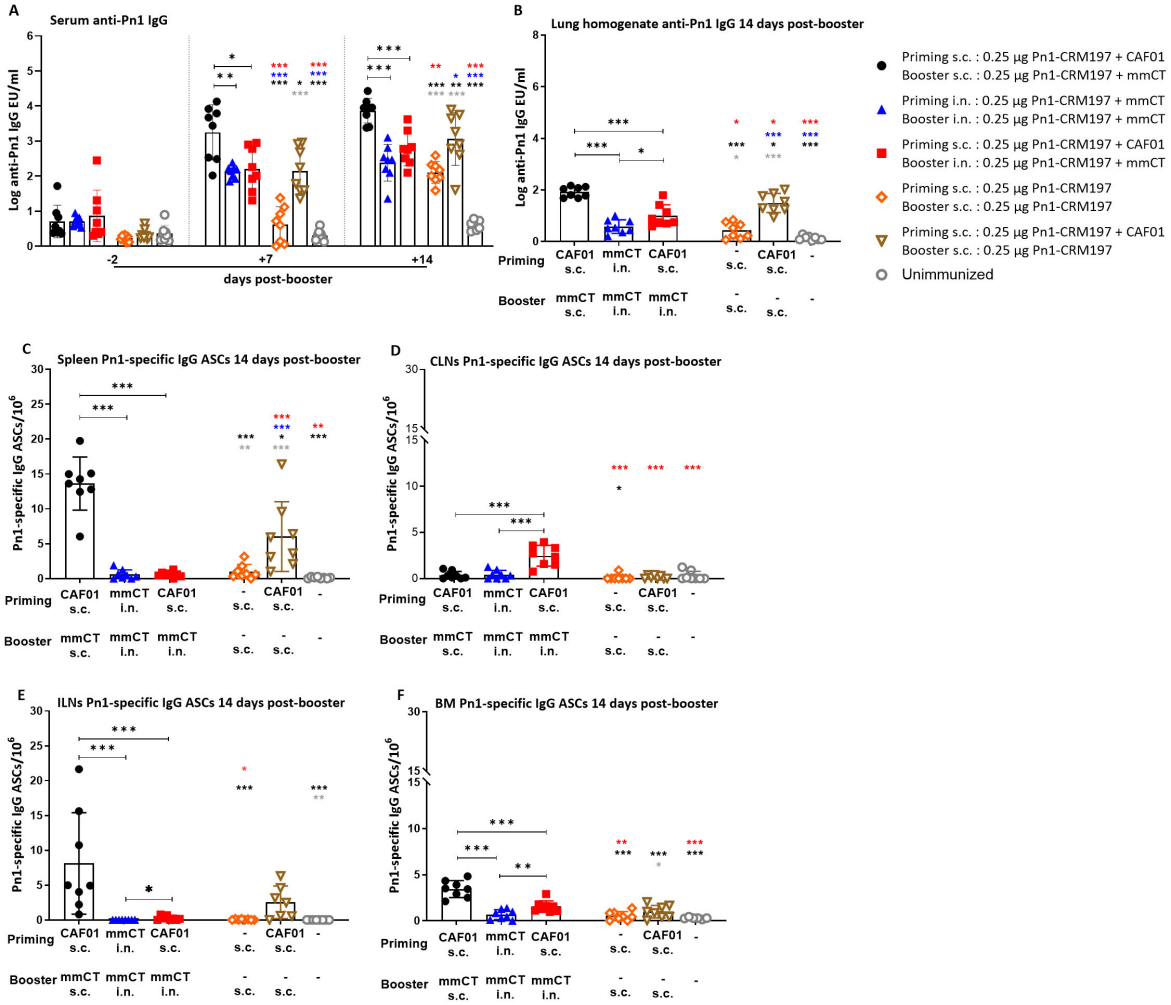
Early-life homologous subcutaneous prime–boost achieves robust anti-Pn1 IgG responses. **(A)** Serum anti-Pn1 IgG levels at −2 to 14 days post-booster. **(B)** Lung homogenate anti-Pn1 IgG levels at 14 days post-booster. **(C–F)** Pn1-specific IgG^+^ ASCs in spleen, CLNs, ILNs, and BM at 14 days post-booster. Mice were immunized by different immunization schedules utilizing 0.25 µg of Pn1-CRM_197_, CAF01, and 2 µg of mmCT. Results are expressed as IgG levels (mean log EU/mL ± SD) or number of spots/10^6^ cells (mean ± SD) in six to eight– mice per group. Statistical difference was calculated using Kruskal–Wallis test first (**A–F**, p < 0.0001), and then Mann–Whitney U-test was applied. *p < 0.05, **p < 0.01, and ***p < 0.001. Comparisons to reference groups are marked on the bars in the corresponding test group colors. ASCs, antibody-secreting cells; BM, bone marrow; mmCT, multiple mutant of cholera toxin.

**Figure 4:**
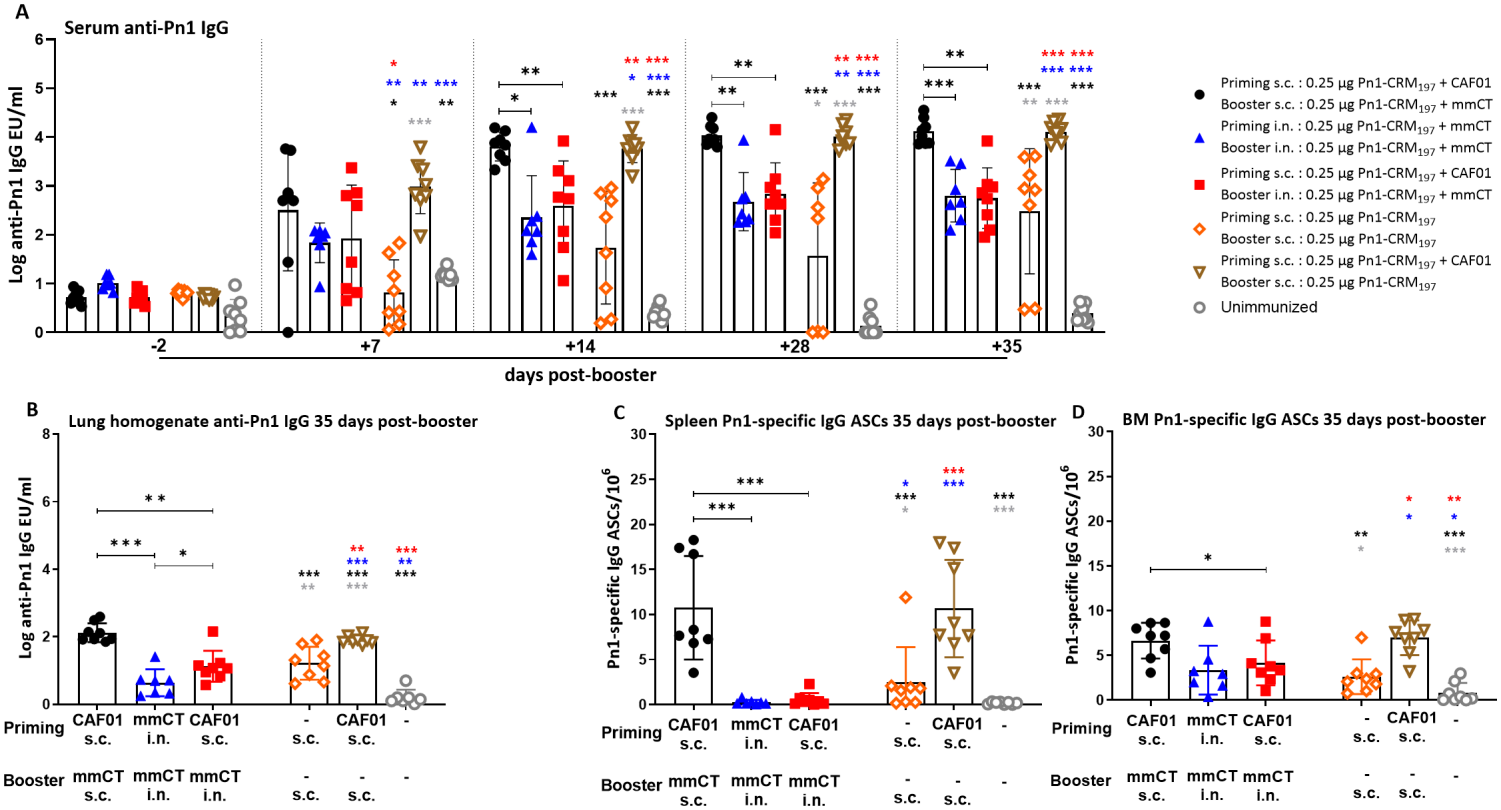
Early-life homologous subcutaneous prime–boost achieves robust anti-Pn1 IgG responses. **(A)** Serum anti-Pn1 IgG levels at −2 to 35 days post-booster. **(B)** Lung homogenate anti-Pn1 IgG levels at 35 days post-booster. **(C, D)** Pn1-specific IgG^+^ ASCs in spleen and BM at 14 days post-booster. Mice were immunized by different immunization schedules utilizing 0.25 µg of Pn1-CRM_197_, CAF01, and 2 µg of mmCT. Results are expressed as IgG levels (mean log EU/mL ± SD) or number of spots/10^6^ cells (mean ± SD) in six to eight– mice per group. Statistical difference was calculated using Kruskal–Wallis test first (**A–D**, p < 0.0001), and then Mann–Whitney U-test was applied. *p < 0.05, **p < 0.01, and ***p < 0.001. Comparisons to reference groups are marked on the bars in the corresponding test group colors. ASCs, antibody-secreting cells; BM, bone marrow; mmCT, multiple mutant of cholera toxin.

Next, we wanted to compare systemic responses induced by heterologous and homologous route prime–boost strategies by measuring Pn1-specific IgG in serum and lung homogenates as well as the induction of Pn1-specific IgG ASCs in the spleen, CLNs, and ILNs; their homing to the BM at 14 days post-booster (**Figure 3**); and persistence at 35 days post-booster (**Figure 4**).

The heterologous s.c./i.n. schedule did not induce as high IgG Ab levels in serum and lung homogenates as the homologous s.c./s.c. schedules. Thus, homologous s.c./s.c. immunized mice consistently had the highest IgG Ab levels in serum and lung homogenates at 7 to 35 days post-booster (**Figures 3A, B**, **4A, B**). Interestingly, heterologous s.c./i.n. immunization induced higher Pn1-specific IgG levels than the homologous i.n./i.n. schedule in lung homogenates at 14 and 35 days post-booster (**Figures 3A, B**, **4A, B**). In agreement, homologous s.c./s.c. immunization induced a higher number of specific IgG ASCs in the spleen (days 14 and 35), ILNs (day 14), and BM (day 14) than both the heterologous s.c./i.n. and homologous i.n./i.n. schedules (**Figures 3C, E, F**, **4C**). Moreover, homologous s.c./s.c. induced higher numbers of IgG ASCs in the BM at 35 days post-booster than heterologous s.c./i.n. (**Figure 4D**). In contrast, heterologous s.c./i.n. induced the highest number of specific IgG ASCs in CLNs (**Figures 3D, F**), as was observed for IgA ASCs (**Figure 1G**). Moreover, heterologous s.c./i.n. immunization accelerated the early homing of specific IgG ASCs to the BM, similarly to IgA ASCs, but only in comparison with the homologous i.n./i.n. schedule (**Figure 3F**), and this difference also diminished with time (**Figure 4D**).

Using a well-established murine model, we have demonstrated in our previous publications that Pn1-specific IgG levels above log 1.5 and log 2.5 EU/mL are protective against pneumococcal bacteremia and lung infection, respectively, when mice are challenged with *S. pneumoniae* serotype 1 (13, 31–33). It should be noted that in this study, infection challenge and protection were not assessed; the protective thresholds were set based on prior studies using a different vaccine formulation, and additional potential correlates of protection may include T-cell responses but were not evaluated here. Accordingly, we calculated the percentage of mice in each group that reached protective Ab levels against bacteremia or lung infection since infection challenge was not feasible in this study. For 87.5%–100% of heterologous s.c./i.n. immunized mice, the IgG levels reached the protective levels against bacteremia and 62.5%–75% against lung infection at 14–35 days post-booster. Of homologous i.n./i.n. immunized mice, 100% had IgG levels that reached the protective levels against bacteremia, but only 14.29%–71.43% of them reached the protective levels against lung infection at 14 to 35 days post-booster. However, all (100%) homologous s.c./s.c. immunized mice had IgG levels that reached the protective levels against both lung infection and bacteremia at 14, 28, and 35 days post-booster (**Figure 4A**, **Supplementary Table 1**).

These results demonstrate that the heterologous s.c./i.n. immunization schedule does not increase the systemic IgG Ab levels or enhance the induction of IgG ASCs to a similar degree as homologous s.c./s.c. Furthermore, the homologous s.c./s.c. schedule induces more systemic (spleen) and local (ILN) humoral immune responses, while heterologous s.c/i.n. immunization induces more local humoral immune responses in draining lymph nodes (CLN), which seems to accelerate the early homing of IgG ASCs to the bone marrow in comparison with homologous i.n./i.n. immunization.

### 3.3 Heterologous immunization utilizing a higher dose of Pn1-CRM_197_ in intranasal booster yields promising Pn1-specific IgG and IgA responses

**Figure 5:**
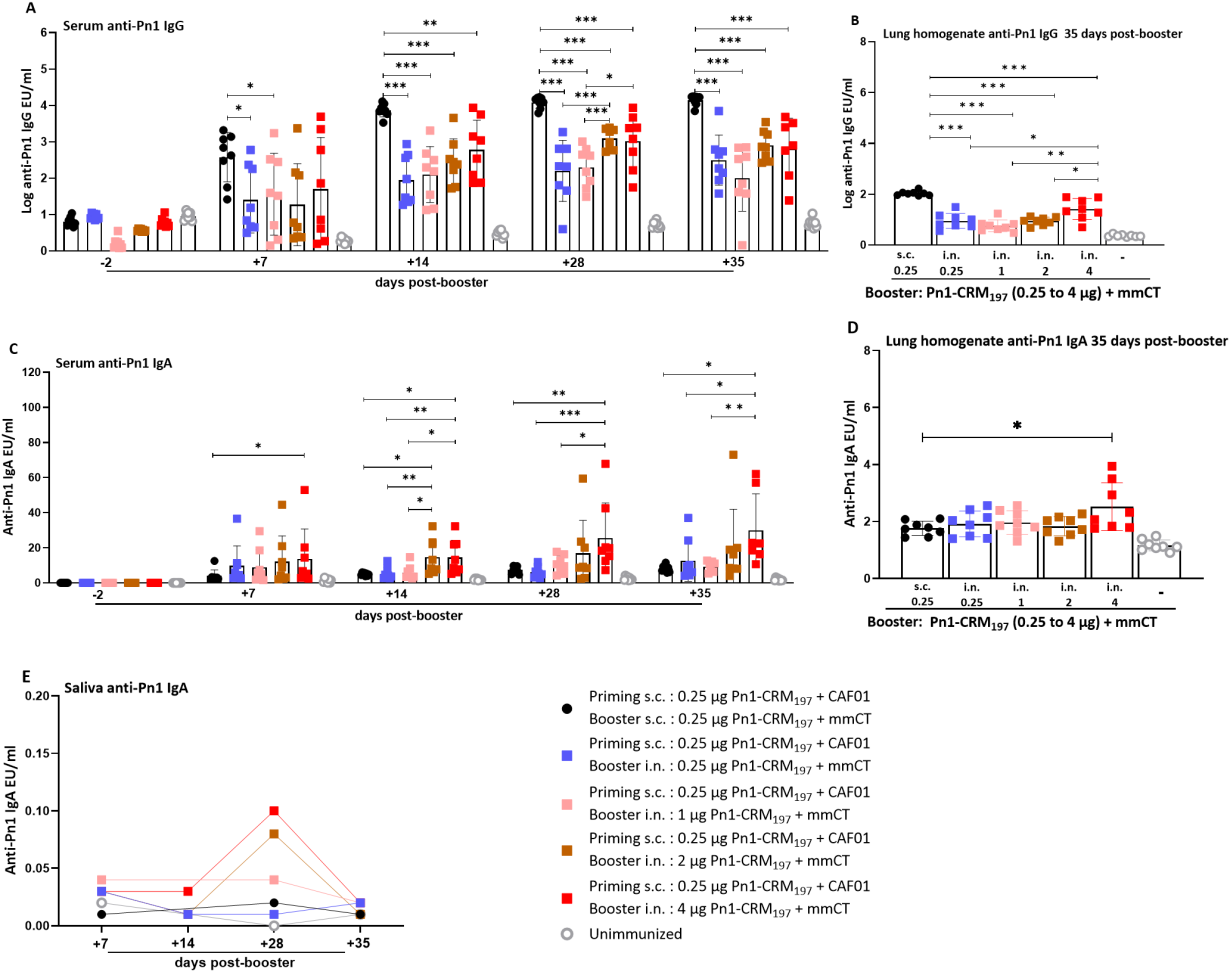
Higher dose of Pn1-CRM_197_ in booster intranasal immunization of heterologous immunization schedule results in higher anti-Pn1 IgG and IgA levels. **(A)** Serum anti-Pn1 IgG levels at −2 to 35 days post-booster. **(B)** Lung homogenate anti-Pn1 IgG levels at 35 days post-booster. **(C)** Serum anti-Pn1 IgA levels at −2 to 35 days post-booster. **(D)** Lung homogenate anti-Pn1 IgA levels at 35 days post-booster. **(E)** Saliva anti-Pn1 IgA levels at 7 to 35 days post-booster. Mice were immunized by different immunization schedules utilizing 0.25, 1, 2, or 4 µg of Pn1-CRM_197_, CAF01, and 2 µg of mmCT. Results are expressed as IgG levels (log mean EU/mL ± SD) or IgA levels (mean EU/mL ± SD) in six to eight– mice per group, and statistical difference was calculated using Kruskal–Wallis test first (**A–C**, p < 0.0001, and **D**, p < 0.0007), and then Mann–Whitney U-test was applied. *p < 0.05, **p < 0.01, and ***p < 0.001. mmCT, multiple mutant of cholera toxin.

We have recently reported that a higher dose of Pn1-CRM_197_ was needed when administered i.n. with mmCT to achieve a comparable IgG response to that elicited by s.c. immunization with Pn1-CRM_197_ and mmCT (12). Therefore, we wanted to assess the effects of increased i.n. booster doses of Pn1-CRM_197_ (0.25, 1, 2, and 4 µg) with mmCT in Pn1-CRM_197_ and CAF01 s.c. primed mice (heterologous s.c./i.n. immunization) compared with homologous s.c./s.c. immunization, the most effective schedule so far in inducing IgG humoral response (**Figures 3**, **4**). None of the heterologous s.c./i.n. immunized groups, irrespective of the Pn1-CRM_197_ dose, were able to increase the IgG Ab levels to a comparable level as the homologous s.c./s.c. schedules (**Figure 5A**). All homologous s.c./s.c. immunized mice had IgG levels that reached the protective levels at 14 to 35 days post-booster (**Figure 5A**, **Supplementary Table 2**), consistent with previous findings (**Figure 4A**, **Supplementary Table 1**).

All mice boosted i.n. with 2- or 4-µg dose of Pn1-CRM_197_ (heterologous s.c./i.n. immunization) had IgG levels that reached the protective levels against bacteremia earlier than mice boosted with 0.25 or 1 µg of Pn1-CRM_197_, i.e., 14 days post-booster. All mice boosted i.n. with 2 µg Pn1-CRM_197_ in heterologous s.c./i.n. immunization had IgG levels that reached the protective levels against lung infection by day 28, declining to 75% by day 35, whereas 75% of mice that received 4 µg booster dose of Pn1-CRM_197_ had IgG levels that reached protective levels at day 28, increasing to 83.3% by 35 days post-booster (**Figure 5A**, **Supplementary Table 2**).

The specific IgG levels in lung homogenate were higher in homologous s.c CAF01/s.c. mmCT immunized mice than all heterologous s.c./i.n. immunization groups at 35 days post-booster. Additionally, the mice that received 4 µg of Pn1-CRM_197_ in the i.n. booster had higher IgG levels in lung homogenate than mice that received lower booster doses in heterologous immunization (**Figure 5B**).

Heterologous s.c./i.n. immunized mice that received 4 µg of Pn1-CRM_197_ in the booster consistently had the highest IgA levels in serum, lung homogenate, and saliva compared to all other immunized groups (**Figures 5C–E**).

**Figure 6:**
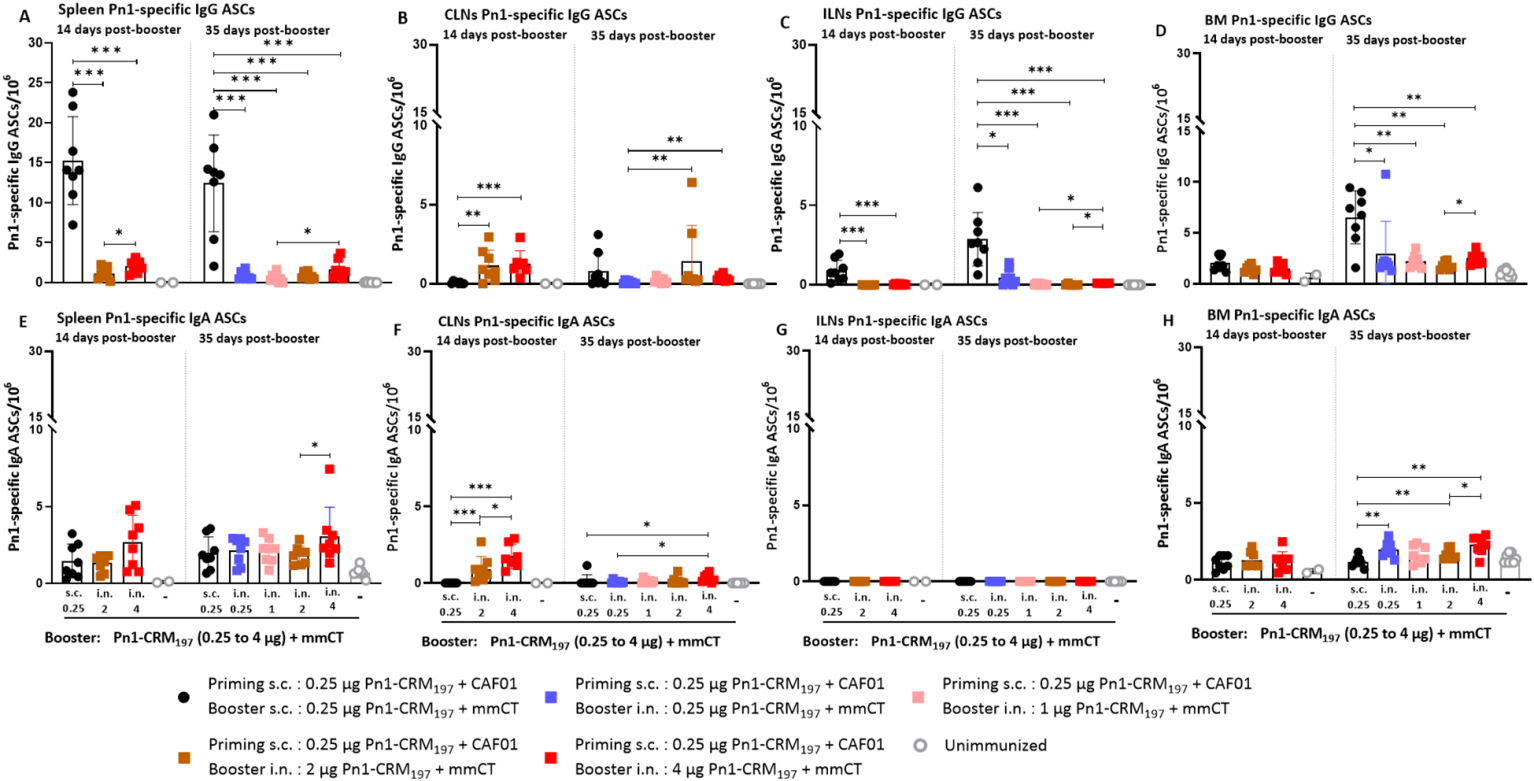
Evaluating the persistence of Pn1-specific ASCs following higher dose of Pn1-CRM_197_ booster intranasal in heterologous immunization schedule. **(A–D)** Pn1-specific IgG^+^ ASCs in spleen, CLNs, ILNs, and BM at 14 and 35 days post-booster. **(E–H)** Pn1-specific IgA^+^ ASCs in spleen, CLNs, ILNs, and BM at 14 and 35 days post-booster. Mice were immunized by different immunization schedules utilizing 0.25, 1, 2, or 4 µg of Pn1-CRM_197_, CAF01, and 2 µg of mmCT. Results are expressed as the number of spots/10^6^ cells (mean ± SD) in six to eight– mice per group, statistical difference was calculated using Kruskal–Wallis test first (**A–D, F, H**, p < 0.0001, and **E**, p < 0.0011), and then Mann–Whitney U-test was applied. *p < 0.05, **p < 0.01, and ***p < 0.001. ASCs, antibody-secreting cells; BM, bone marrow; mmCT, multiple mutant of cholera toxin.

In line with previous results, heterologous s.c./i.n. immunized mice that received higher booster doses of Pn1-CRM_197_ had a higher number of specific IgG ASCs in CLNs than homologous s.c./s.c. immunized mice at 14 days but not at 35 days post-booster (**Figure 6B**). Accordingly, homologous s.c./s.c. immunized mice had the highest number of specific IgG ASCs in the spleen and ILNs at 14 and 35 days post-booster and in the BM at 35 days post-booster (**Figures 6A, C, D**).

When IgA responses were assessed, no difference in the number of specific IgA ASCs in the spleen was observed between heterologous s.c./i.n. and homologous s.c./s.c. immunized mice at 14 and 35 days post-booster (**Figure 6E**). Mice immunized with 4 µg of Pn1-CRM_197_ in the i.n. booster had the highest number of specific IgA ASCs in CLNs at 14 and 35 days post-booster (**Figure 6F**), while no IgA ASCs were detected in ILNs (**Figure 6G**). The number of specific IgA ASCs in the BM was generally higher in heterologous s.c./i.n. immunized mice than homologous s.c./s.c. immunized mice at 35 days post-booster (**Figure 6H**).

Overall, even though increasing the vaccine dose in the i.n. booster resulted in higher and more persistent IgA levels and a greater percentage of mice had serum IgG levels that reached the protective levels against lung infection and bacteremia, the IgG response was still not comparable to the response induced by the homologous s.c./s.c. schedule.

## 4 Discussion

Heterologous immunizations involving different vaccine types, delivery routes, adjuvants, or formulations for priming and boosting have gained significant attention in recent years (14). Our group has demonstrated that i.n. immunization with Pnc1-TT and LT-K63 in neonatal and infant mice can be superior to parenteral immunization in eliciting protective Abs by a single vaccine dose (9, 13); however, clinical trials have shown safety concerns, and therefore, the potential of mucosal application with this molecule was reconsidered (45). In neonates, CAF01 can induce bona fide GC responses along with robust and prolonged primary humoral responses in murine neonates, although not to the same extent as in adult mice (27). We have shown in previous studies that mmCT co-administered with the Pn1-CRM_197_ vaccine can enhance the GC formation after both s.c. and i.n. immunizations (12). Mucosal immunization is aimed at protecting against infections at the mucosal level by reducing nasopharyngeal carriage and subsequently invasive diseases (20, 33, 46). IgA antibodies are critical for early defense against respiratory infections, and the passive transfer of IgA can protect against reinfection with those pathogens (47). IgA can also cross-link bacteria and halt their growth (19, 20). IgA and sIgA have been shown to be important in protection against pneumococcal colonization since pIgR^−/−^ mice, which lack the ability to secrete IgA to the mucosal lumen, were not protected like wild-type mice against infection by serotype 14 *S. pneumoniae*, even though they elicited comparable systemic Abs as wild-type mice after i.n. conjugate immunization. Likewise, IgA^−/−^ mice had no protection against colonization (18).

Our study examines the effects of adjuvants CAF01 and mmCT on humoral immune responses in various immune compartments after early-life vaccination through both heterologous and homologous immunization routes.

We found that the immunization route significantly influences the outcome, with i.n. immunization either heterologous s.c./i.n. or homologous i.n./i.n. being more effective than the homologous s.c./s.c. schedule in inducing IgA responses. However, IgA levels in serum and lung homogenates declined over time after both schedules (heterologous s.c./i.n. and homologous i.n./i.n.); in contrast, the persistence of salivary IgA seemed to be better after homologous i.n./i.n. than heterologous s.c./i.n. immunization. Although the homing of vaccine-specific IgA ASCs to the BM seemed to be accelerated by the heterologous s.c./i.n. early post-booster in comparison with homologous i.n./i.n., over time, this difference diminished, as both schedules led to a comparable persistence of specific IgA ASCs in the BM. The ideal outcome of early-life immunization strategies would be the induction of balanced IgA and IgG responses, as IgG Abs have been shown to be the most effective in protecting against invasive infections by encapsulated bacteria like *S. pneumoniae* and have been used as the basis for the licensure of pneumococcal vaccines (16, 17). The homologous route s.c./s.c. schedule induced stronger vaccine-specific IgG responses and more IgG ASCs in the spleen and ILNs, with better persistence in the BM than any of the other strategies. Thus, homologous s.c./s.c. immunization yielded protective serum IgG levels against lung infection and bacteremia in all mice early post-booster (**Supplementary Table 1**). Of note, heterologous s.c./i.n. immunization yielded the highest numbers of Pn1-specific IgA and IgG ASCs in CLNs (**Figures 1**, **3**) and the earlier homing of IgA ASCs to the BM than any other immunization schedule (**Figure 2**). This was also observed for IgG ASC but only in comparison with homologous i.n./i.n. immunization. In a clinical trial, the chlamydia vaccine candidate, CTH522, was tested and given with CAF01 as an adjuvant three times intramuscularly (i.m.), and then the subjects received two i.n. booster with only the vaccine. This study showed that combining parenteral and mucosal immunizations as a heterologous prime–boost strategy generated strong vaccine-specific IgG and some IgA responses in which i.n. boosters elicited stronger mucosal vaccine-specific responses but had no enhancing effect on IgG responses in comparison with parenteral application (48).

Interestingly, we found that with our heterologous immunization design, we obtained higher vaccine-specific IgG responses than homologous i.n./i.n. in different immune compartments, including IgG levels in lung homogenates at 14 and 35 days post-booster (**Figures 3**, **4**) and stronger induction of IgG ASCs in CLNs and ILNs at 14 days post-booster (**Figure 3**). Furthermore, s.c./i.n. had earlier IgG ASC homing to the BM than i.n./i.n. immunization (**Figure 3**). However, homologous i.n./i.n. immunization induced a systemic humoral response seen by the induction of ASC, mainly in the spleen, but also enhanced mucosal salivary and lung IgA Abs.

Mucosal immunization often requires the use of adjuvants and/or specialized delivery systems to elicit antigen-specific immunity due to challenging mucosal environments for vaccines mediated by, e.g., digestive enzymes, mucus secretion, and ciliary movement (49). Since our prior study showed that higher i.n. doses of Pn1-CRM_197_ with mmCT in a single immunization were necessary to induce comparable IgG responses to those elicited by s.c. immunization (12), we examined the effect of increasing the dose of Pn1-CRM_197_ in the i.n. booster with the heterologous s.c./i.n. schedule.

Increasing the dose of Pn1-CRM_197_ in the i.n. booster yielded higher specific IgA levels in serum, saliva, and lung homogenates (**Figure 5**) and increased the proportion of mice reaching the protective levels of specific IgG Abs against bacteremia (**Supplementary Table 2**). However, increased doses of Pn1-CRM_197_ in the i.n. booster did not result in comparable IgG anti-Pn1 levels in serum and lung homogenates as elicited by homologous s.c./s.c. immunization.

We described above that IgG Abs are crucial for protecting against invasive infections by encapsulated bacteria like *S. pneumoniae* and are the foundation for pneumococcal vaccine approval. It is unclear whether the lower levels of vaccine-specific IgG observed with heterologous s.c./i.n. immunization compared to homologous s.c./s.c. negatively impacted protection in our mouse infection model. This uncertainty arises because the higher levels of vaccine-specific IgA induced by the heterologous approach may effectively limit the pathogen’s spread in the respiratory system and prevent further invasion and disease. In our model, we have shown that one i.n. immunization with a pneumococcal conjugate vaccine induces better protective immunity against a pneumococcal infection than parenteral immunization in neonatal mice, especially against lung infection, and is associated with a high salivary IgA response (9). It has also been shown that in another pneumococcal infection model using adult Balb/c mice vaccinated with pneumococcal protein PSP, both parenteral and oral vaccinations provided similar protection against infection, as measured by lung bacterial load and mortality rates. Noteworthy, though, was that parenteral immunization induced a faster and stronger PSP-specific IgG response than the oral immunization, which was not translated to a better protection compared to oral immunization, which achieved comparable efficacy despite lower serum IgG levels at the time of infection (50), strongly supporting the benefits of mucosal IgA Abs.

Taken together, this study explores whether heterologous route immunization strategies provide any advantages in the induction of humoral immune response during early life. These findings support the potential of heterologous s.c./i.n. immunization in early life, as it elicited stronger vaccine-specific IgG responses in certain immune compartments compared to homologous i.n./i.n. immunization without compromising IgA responses. However, further studies are needed for better optimizations and assessments. We demonstrated that homologous s.c./s.c. immunization induces higher systemic specific IgG responses than heterologous s.c./i.n. immunization, even when the i.n. booster dose of Pn1-CRM_197_ was increased. This study shows the importance of strategic use of adjuvants, vaccine dosage assessments, and homologous and heterologous routes of immunizations in order to improve vaccine efficacy against respiratory pathogens.

## Data Availability

The raw data supporting the conclusions of this article will be made available by the authors, without undue reservation.
