# Supplementary material for: Distinct humoral responses induced by heterologous versus homologous prime–boost immunization strategies in early life
Source: Front Immunol. 2025 Aug 7;16:1563345. doi: 10.3389/fimmu.2025.1563345 (PMC12367788; doi:10.3389/fimmu.2025.1563345)
Supplement: Supplementary file 1 [file DataSheet1.docx]

Supplementary Material

Heterologous prime-boost immunization routes strategy in early-life to enhance protective immunity against respiratory pathogens

Poorya Foroutan Pajoohian^1,2^, Audur Anna Aradottir Pind^1,2^, Jenny Lorena Molina Estupiñan^1,2^, Dennis Christensen^3^, Gabriel Kristian Pedersen^3^, Thorunn A. Olafsdottir^1^, Ingileif Jonsdottir^1,2^, Stefania P. Bjarnarson^1,2*^

^1^Faculty of Medicine, Biomedical Center, School of Health Sciences, University of Iceland, Reykjavik, Iceland

^2^Department of Immunology, Landspitali, the National University Hospital of Iceland, Reykjavik, Iceland

^3^ Center for Vaccine Research, Statens Serum Institut, Copenhagen, Denmark

*** Correspondence:**Stefania P. Bjarnarson
stefbja@landspitali.is

# Supplementary Figures and Tables

## Supplementary Figures


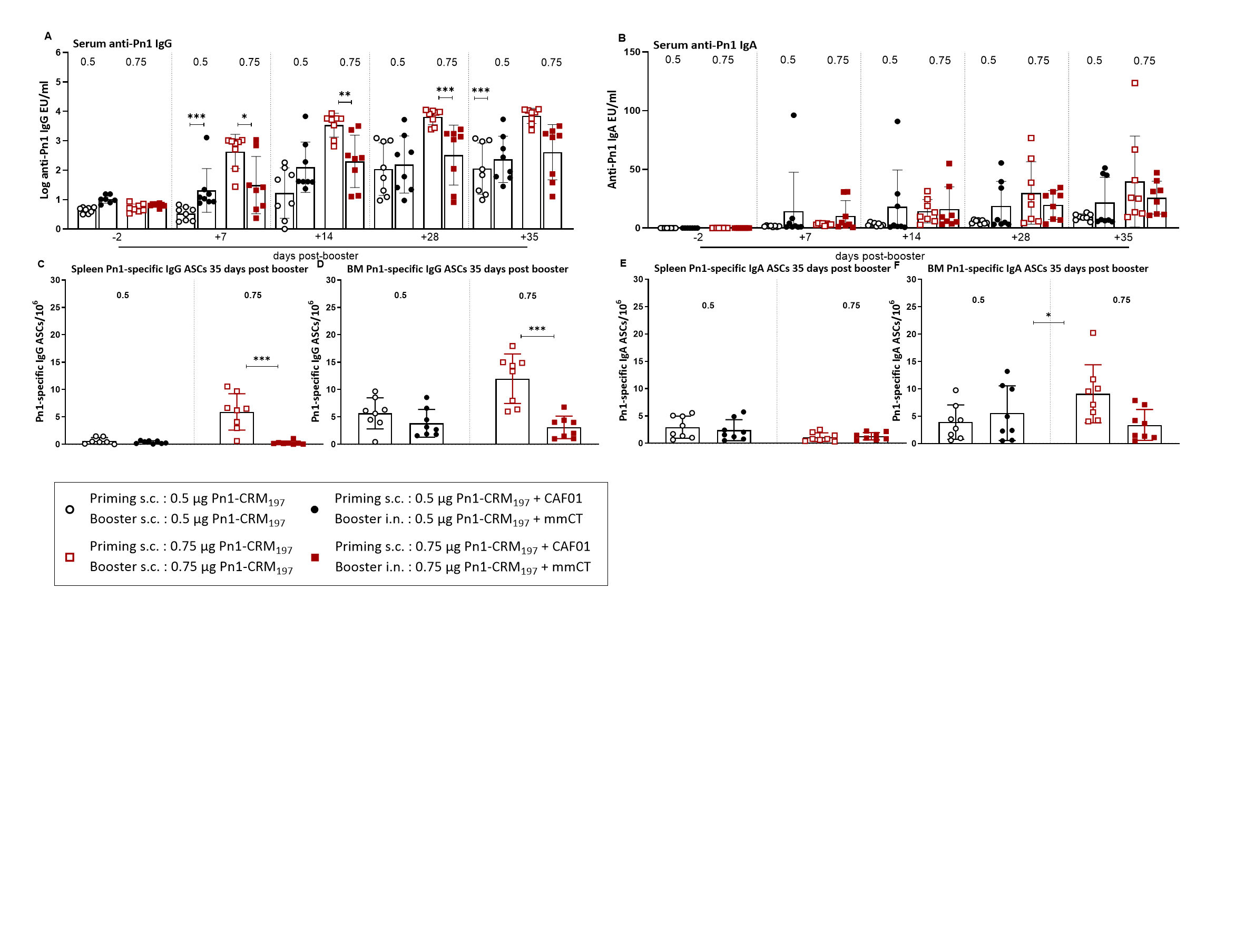


**Supplementary Figure 1**. **Finding the optimal dose of Pn1-CRM_197._** (A-B) Serum IgG/IgA levels -2 to 35 days post booster. (C-F) IgG+/IgA+ Pn1-specific ASCs in spleen and BM 35 days post-booster. Mice were immunized by different immunization schedules utilizing 0,5 and 0,75 µg of Pn1-CRM197 with or without CAF01, and 5 µg of mmCT. Results are expressed as IgG levels (mean log EU/ml ± SD), IgA levels (mean EU/ml ± SD), or number of spots/10^6^ cells (mean ± SD) in 6-8 mice per group. Statistical difference was calculated using Mann–Whitney U-test. *p < 0.05, **p < 0.01, ***p < 0.001.


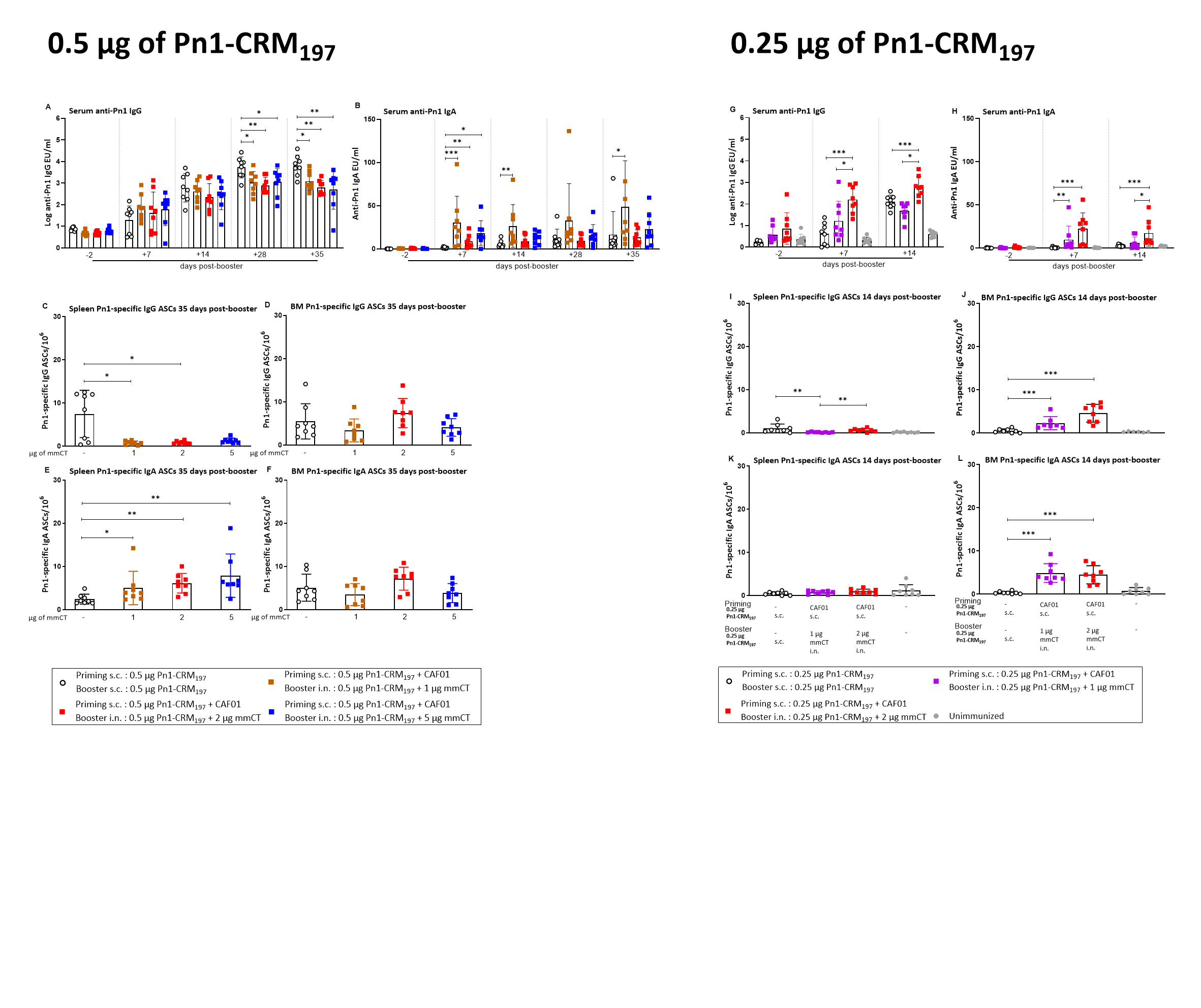


**Supplementary figure 2. Finding the optimal dose of Pn1-CRM_197_ and optimal dose of mmCT.**

(A-B) Serum IgG/IgA anti-Pn1 levels -2 to 35 days post-booster utilizing 0.5 µg of Pn1-CRM197 and different doses of mmCT (1,2, and 5 µg) for intranasal booster. (C-F) IgG^+^/IgA^+^ Pn1-specific ASCs in spleen and BM 35 days post-booster. (G-H) Serum IgG/IgA anti-Pn1 levels -2 to 14 days post-booster utilizing 0.25 µg of Pn1-CRM197 and different doses of mmCT (1 and 2 µg) for intranasal booster. (I-L) IgG^+^/IgA^+^ Pn1-specific ASCs in spleen and BM 14 days post-booster. Mice were immunized by different immunization schedules utilizing 0.25 and 0.5 µg of Pn1-CRM _197_ with or without CAF01, and mmCT. Results are expressed as IgG levels (mean log EU/ml ± SD), IgA levels (mean EU/ml ± SD), or number of spots/10^6^ cells (mean ± SD) in 6-8 mice per group. Statistical difference was calculated using Mann–Whitney U-test. *p < 0.05, **p < 0.01, ***p < 0.001.

## Supplementary Tables

**Supplementary Table 1. Percentage of protective anti-Pn1 IgG antibody levels against bacteremia and lung infection -2 to 35 days post-booster.** Mice were immunized by different immunization schedules utilizing 0.25µg of Pn1-CRM _197_, CAF01, and 2µg of mmCT in 6-8 mice per group.

**Supplementary Table 2. Percentage of protective anti-Pn1 IgG antibody levels against bacteremia and lung infection -2 to 35 days post-booster after increasing intranasal booster dose of Pn1-CRM_197_.** Mice were immunized by different immunization schedules utilizing 0.25, 1, 2, and 4µg of Pn1-CRM _197_, CAF01, and 2µg of mmCT.
